# Diapause-Linked Gene Expression Pattern and Related Candidate Duplicated Genes of the Mountain Butterfly *Parnassius glacialis* (Lepidoptera: Papilionidae) Revealed by Comprehensive Transcriptome Profiling

**DOI:** 10.3390/ijms24065577

**Published:** 2023-03-14

**Authors:** Chengyong Su, Chen Ding, Youjie Zhao, Bo He, Ruie Nie, Jiasheng Hao

**Affiliations:** College of Life Sciences, Anhui Normal University, Wuhu 241000, China

**Keywords:** Transcriptome, *Parnassius glacialis*, diapause, environmental adaptation, genome

## Abstract

The mountain butterfly *Parnassius glacialis* is a representative species of the genus *Parnassius,* which probably originated in the high-altitude Qinhai–Tibet Plateau in the Miocene and later dispersed eastward into relatively low-altitude regions of central to eastern China. However, little is known about the molecular mechanisms underlying the long-term evolutionary adaptation to heterogeneous environmental conditions of this butterfly species. In this study, we obtained the high-throughput RNA-Seq data from twenty-four adult individuals in eight localities, covering nearly all known distributional areas in China, and firstly identified the diapause-linked gene expression pattern that is likely to correlate with local adaptation in adult *P. glacialis* populations. Secondly, we found a series of pathways responsible for hormone biosynthesis, energy metabolism and immune defense that also exhibited unique enrichment patterns in each group that are probably related to habitat-specific adaptability. Furthermore, we also identified a suite of duplicated genes (including two transposable elements) that are mostly co-expressed to promote the plastic responses to different environmental conditions. Together, these findings can help us to better understand this species’ successful colonization to distinct geographic areas from the western to eastern areas of China, and also provide us with some insights into the evolution of diapause in mountain *Parnassius* butterfly species.

## 1. Introduction

In the context of global climatic changes throughout Earth’s history, insects in the field are usually exposed to repeated bouts of stress (e.g., cold/heat, dry/moist and solar radiation) and unpredictable factors (e.g., predators, pathogenic microorganisms, food supply and population density), under the varied intensity or amplitudes of fluctuating conditions [1,2]. Correspondently, determining the drivers and the resultant patterns of gene expression is more complicated in the fluctuating environments where insects typically live than in controlled laboratory conditions. For example, previous studies showed that both the mean and fluctuation of temperature could contribute to thermal acclimation and affect the transcriptional pattern in *Drosophila melanogaster* [3]; repeated and single cold factors could induce divergent transcriptomic responses [1]; photoperiodism might mediate insect phenological responses to temperature [4], and the synergistic effects of multiple stressors could induce novel candidate genes responsible for the variation found in thermal tolerance and survival [5,6,7]. However, insects have evolved to overcome unfavorable environmental conditions in a hormonally regulated state of diapause, during which their activity is suppressed and their development is decelerated, but their tolerance of environmental stress is bolstered (e.g., increased stress resistance, improved immune defense and somatic maintenance) [8,9,10,11], reflecting their shared transcriptional strategies for regulating the hallmark diapause-linked physiological phenotypes, especially at the functional pathway level [11]. Nevertheless, our knowledge of butterflies’ (including those of new colonizers or nonnative invaders) responses to the changes of natural conditions for survival in the field remains limited.

**Figure 1:**
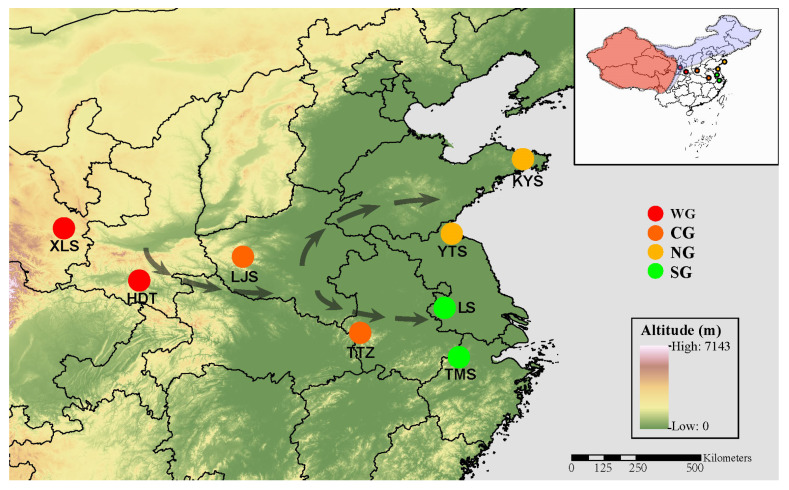
Geographic distribution of *Parnassius glacialis* from eight sampling localities. The proposed dispersal pattern is shown with arrows. WG, western group; CG, central group; NG, northeastern group; and SG, southeastern group (similarly hereinafter). Main distribution areas of other species of the genus *Parnassius* with high (in red) and low diversity (in light blue) in China and adjacent regions are shown in the upper right.

The genus *Parnassius* is a typical mountain-adapted butterfly group, mainly distributed across the Holarctic, with its highest diversity on the Qinghai–Tibet Plateau (QTP) and adjacent mountainous regions (including Xinjiang and Gansu, China), with a broad elevational range of 3000–5000 m (Figure 1). Previous studies have indicated that the diversification of *Parnassius* initiated during the Middle Miocene, correlated with their host plant’s spatiotemporal distributions, and geological and paleoenvironmental changes in the QTP region [12,13], as well as the fact that both the ancient gene introgression and climate cooling after the Middle Miocene Climate Optimum (MMCO) might have contributed to the spread of *Parnassius* species to different altitudes, accompanied by the dispersal from West China to Northeast China and other areas of East Asia [14]. Among them, the Glacial Apollo butterfly, *Parnassius glacialis*, is the only species that has dispersed into the southeastern areas of the Yangtze River, and mainly inhabits low-altitude mountains (~200 to 1800 m), suggesting their extraordinary flexibility to local seasonal environmental challenges. Previous studies have demonstrated that *P. glacialis* diverged firstly into two clades during the Pleistocene period, then dispersed independently into distinct geographic areas from the western to eastern China, most likely driven by the Pleistocene’s glacial–interglacial cycles [15]. Currently, most *P. glacialis* populations are restricted to ecologically and topographically fragmented habitats, especially in relatively lower-altitude mountains in the northeast and southeast of China, with remarkable morphological adaptations, such as body size enlargement and wing color lightening, implying that they have adapted locally to disparate ecological zones. Thus, these populations could offer an excellent butterfly model to examine the intraspecific transcriptional variation in the field, and how this would influence climate change-driven phenotypes.

In the present study, we sampled a total of 24 *P. glacialis* individuals from eight localities, and determined large scale transcriptomic data to identify the common genome-wide transcriptomic expression pattern, as well as their intraspecific transcriptional variations. Meanwhile, we attempted to reveal the distinctive transcriptomic signatures of duplicated genes and transposable elements (TEs), based on the newly sequenced high-quality genome of *P. glacialis* from our laboratory, in order to deepen our understanding of the “out of QTP” dispersal and adaptation to different environmental conditions of *P. glacialis*.

## 2. Results

### 2.1. Statistics of Differentially Expressed Genes (DEGs) and Functional Enrichment Analysis

In order to dissect the molecular mechanisms underlying *P. glacialis’* adaptation to different natural habitats, a total of 24 samples in four groups were used for RNA-seq analysis (Figure 1), and 6.4–10.3 Gb of clean data for each sample were obtained (Table S1). The boxplots show the normalized gene expression profiles and principal component analysis (PCA) scatter plots show differences among samples dwelling in different habitats (Figure S1).

**Figure 2:**
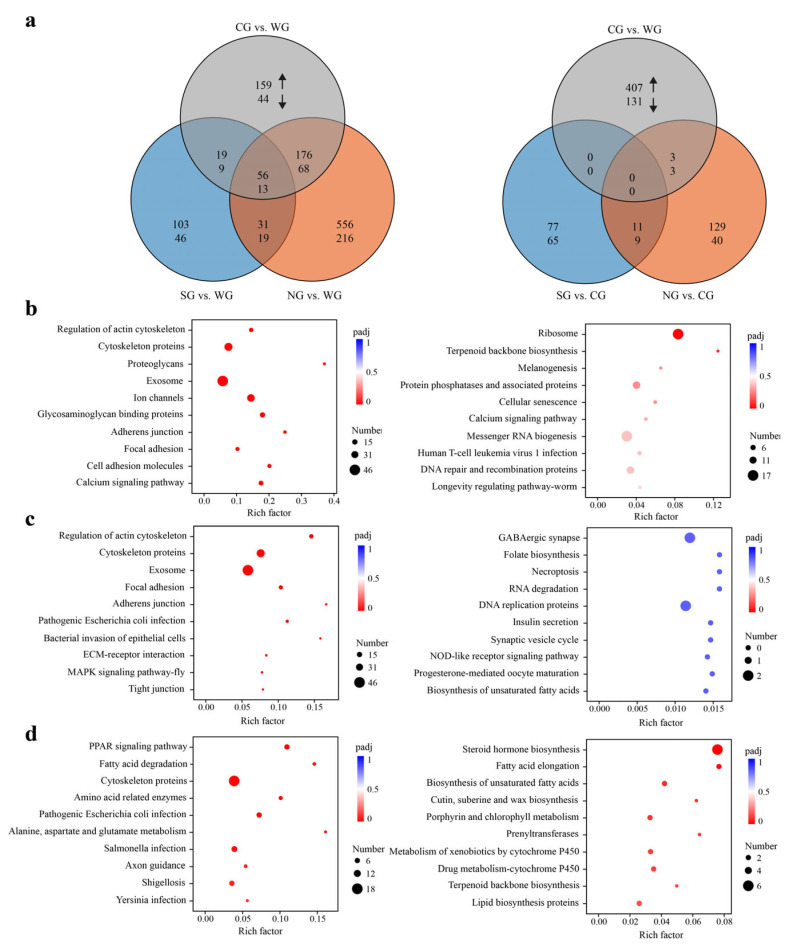
Overview of transcriptional changes among groups based on differential expression analysis. (**a**) Venn diagrams showing overlaps of DEGs with increased (upward arrow) or decreased (downward arrow) transcript abundance in five pairs of comparisons. (**b**–**d**) KEGG enrichment results of the DEGs (left: up-regulated; right: down-regulated) in pairwise comparisons of NG vs. WG, CG vs. WG, and SG vs. WG, respectively. For each comparison, only the top ten pathways with the most significant enrichment are shown.

When compared to the WG samples, the quantitative aspect of the transcriptional changes, roughly judged by comparing the DEGs numbers, gradually decreased from the NG samples to the CG and SG samples, i.e., for NG vs. WG, 6.8% (1,135) of the genes from a total 16,659 sequences were DEGs; for CG vs. WG—3.3% (544), and for SG vs. WG—1.8% (296), probably suggesting that the differences in gene expression were not significantly correlated with geographical distances between WG and other groups. When compared to the CG samples, the decreased DEG numbers were also found for the NG samples (1.2%, 195 for NG vs. CG) and the SG samples (1.0%, 162 for SG vs. CG), but both of these had markedly fewer DEGs than were found in CG vs. WG (3.3%, 544). Moreover, the qualitative aspect of transcriptional changes also tends to evolve gradually, as indicated by the gradual decrease in overlap of DEGs between comparisons, i.e., for NG and WG vs. CG and WG, 23.0% (313) of 1,366 DEGs overlap, whereas for SG and WG vs. CG and WG, only 13.1% (97) of 743 DEGs overlap. Further, for CG and WG vs. NG and CG, 0.8% (6) of 733 DEGs overlap, whereas for CG and WG vs. SG and CG, no DEGs overlap (Figure 2a).

KEGG enrichment analyses showed consistent trends with those above in both the quantitative and qualitative aspects of transcriptional changes, i.e., gradually decreased number (64, 21 and 10) of enriched signaling pathways for DEGs in the comparisons of NG vs. WG, CG vs. WG, and SG vs. WG, respectively, as well as relatively lower overlap of enriched pathways between comparisons, i.e., for NG and WG vs. CG and WG, 21.4% (15) of 70 pathways overlap, whereas for SG and WG vs. CG and WG, only 10.7% (3) of 28 pathways overlap. There were no overlapped pathways among the comparisons of CG vs. WG, NG vs. CG, and SG vs. CG (Figure S2).

When compared to WG samples, the KEGG enrichment analyses showed that NG samples harbored sixty-three significantly enriched pathways for the up-regulated DEGs, while they harbored only one (ribosome) for the down-regulated DEGs (adjusted *p* value < 0.05; Figure 2b, Tables S3 and S4). The pathways enriched for up-regulated DEGs mainly included proteoglycans, calcium signaling, phototransduction, olfactory transduction, cytoskeleton and immune-related pathways. In addition, components of the pathways related to the endocrine system (e.g., GnRH, insulin, relaxin and other hormone-related signaling pathways) and the central nervous system (CNS, including cholinergic, dopaminergic, synaptic vesicle cycle and long-term potentiation) were also enriched (Table S3) [16,17,18]. These KEGG pathways were mainly involved in cell proliferation, motility, immune regulation, hormone biosynthesis, neural plasticity and responses to environmental stimuli, which are necessary for insect survival, growth and longevity regulation [8,16,19,20,21,22,23,24,25]. In the CG samples, twenty-one KEGG pathways were enriched for up-regulated DEGs, including cytoskeleton, proteoglycans and immune-related pathways, in response to pathogenic infection (Figure 2c and Table S3), fifteen of which were shared with those in the NG samples. No KEGG pathways were significantly enriched for down-regulated DEGs of CG samples (Table S4). For the SG samples, eight and two KEGG pathways were significantly enriched for up- and down-regulated DEGs, respectively (adjusted *p* value < 0.05; Figure 2d, Tables S3 and S4). As regards the up-regulated DEGs, the enriched pathways mainly included PPAR (peroxisome proliferator-activated receptor), fatty acid degradation, cytoskeleton proteins, amino acid metabolism and immune response pathways related to bacterial infection, whereas they were related to lipid metabolism and steroid hormone biosynthesis for down-regulated DEGs. Taken together, these results indicate, that compared to WG samples, the NG, CG and SG samples commonly harbored enriched cytoskeleton and immune-related pathways, with each group also exhibiting separate habitat-specific expression patterns.

### 2.2. General Statistics of Enriched KEGG Pathways Based on GSEAs

**Figure 3:**
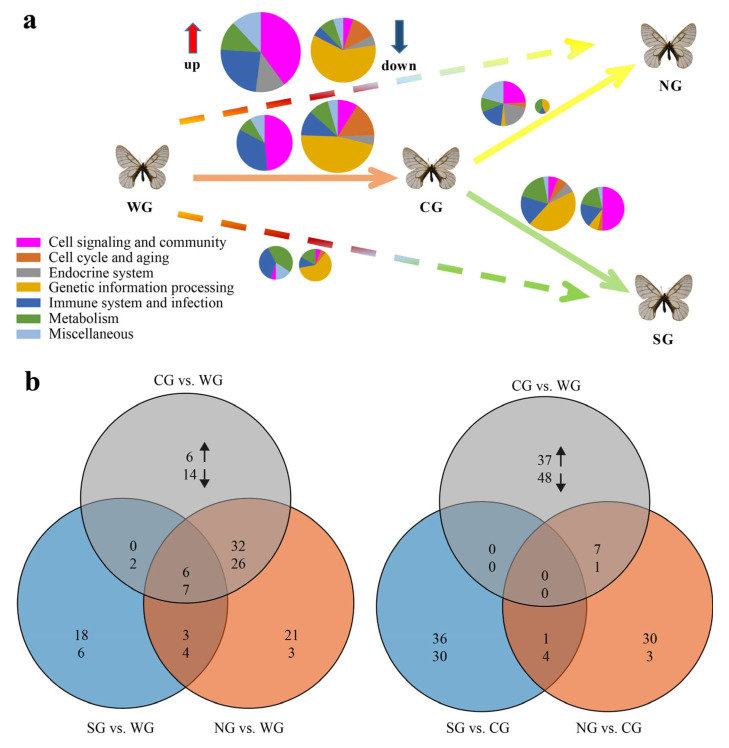
Overview of enriched KEGG pathways based on gene set enrichment analysis. (**a**) The pie charts indicating quantitative (shown by the relative size of the pie) and qualitative (colors of sectors) aspects of the transcriptional change (up-regulation, left pie; down-regulation, right pie) resulted from the long-term evolutionary adaptations to different habitats following the dispersal eastwards. The size of each pie is directly proportional to the percentage of enriched KEGG pathways (excluding those related to human diseases) detected for the respective comparison (e.g., CG vs. WG). The color of each sector codes for the high-level functional category (for more details, see Table S5). (**b**) Venn diagrams showing overlaps of enriched KEGG pathways enhanced (upward arrow) or inhibited (downward arrow) in five pairs of comparisons.

In order to better extract biological insights from the genome-wide expression patterns, multiple GSEAs at both the KEGG pathway and KEGG orthology (KO) levels were conducted, using the normalized and non-normalized datasets. The quantitative aspect of the enriched KEGG pathways can be judged by comparing the sizes of the pie charts (the bigger the pie, the larger the proportion of enriched pathways). The colors of sectors, representing the higher-level functional categories defined according to the KEGG database (https://www.genome.jp/kegg/ (accessed on 16 September 2022)) and the previous study [26], can help to visually identify the changes in gene functional categories and allow a rough comparison of the qualitative aspects of the transcriptional change (Figure 3 and Table S5). In the present study, both the normalized and non-normalized datasets yielded very similar results (Tables S5 and S6). Nonetheless, non-normalized datasets generally resulted in more enriched pathways than normalized ones, especially for the pairwise comparisons of NG vs. WG and NG vs. CG (Table S6), probably due to batch effects (Figure S1), suggesting that the data normalization procedure is necessary.

Overall, our GSEA results corroborate the magnitude and complexity of the transcriptional changes revealed by DEGs and the functional enrichment analyses above. The gradually reduced sizes of pie charts, reflecting the decreasing number of enriched pathways (Figure 3a,b; for NG vs. WG, 102; CG vs. WG, 93; and SG vs. WG, 46), suggest the lack significant correlation between differences in gene expression and geographical distance among compared groups, as shown in the DEG and functional enrichment analyses. Interestingly, the presence of reduced overlap in enriched pathways between comparisons (i.e., for NG and WG vs. CG and WG, 57.3% (71) of 124 pathways overlap, whereas for SG and WG vs. CG and WG, only 12.2% (15) of 123 pathways overlap) possibly indicates that transcriptional changes in SG samples qualitatively differed from those in CG and NG samples, as these groups dispersed eastwards from western area of China [15]. The overall statistics of DEGs, KEGG enrichment analyses and multiple GSEAs consistently suggest that the genome-wide expression pattern of the CG samples was more similar to that of NG samples than to that of the SG samples. In addition, more genes and signaling pathways were probably involved in local environmental adaptation when *P. glacialis* initially dispersed from the western (WG) to central (CG) areas of China, followed by less recruitment of potentially new gene sets for the successive colonization of northeastern (NG) and southeastern (SG) areas, respectively (Figure 3a). 

### 2.3. Featured Gene Sets Based on GSEAs

**Figure 4:**
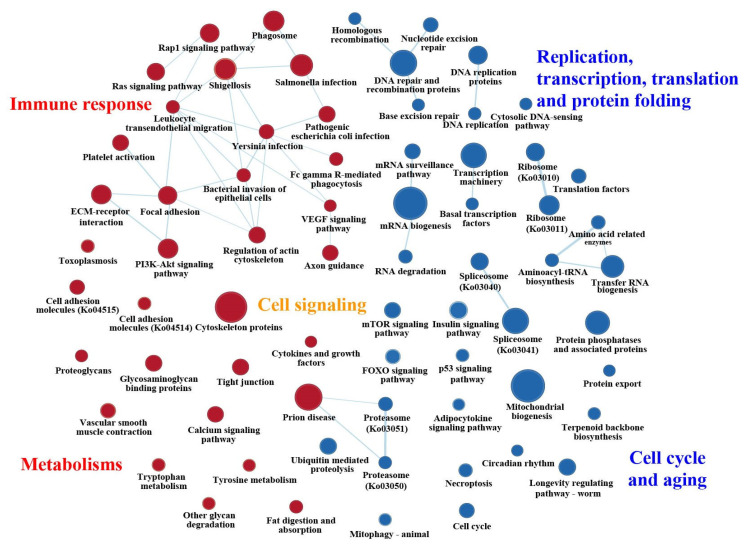
Enrichment map for the shared pathways in at least two groups out of NG, CG and SG in comparison to WG. Circles in red and blue show enhanced and inhibited pathways, respectively, with the size indicating the number of genes belonging to each KEGG pathway. The line thickness, which represents the degree of overlap between two pathways, is shown in light blue. More detailed information is available in Table S5.

In pairwise comparisons to WG samples, the GSEA results based on genome-wide expressed genes reveal that the pathways enriched for mostly up-regulated genes in NG, CG and SG samples are mainly involved in cell signaling, immune system and metabolism, while those enriched for down-regulated genes are markedly related to genetic information processing, cell cycle and aging, regardless of datasets used (Figure 3a and Table S5). Specifically, enhanced pathways, including cytoskeleton proteins, focal adhesion, tight junction, proteoglycans, calcium signaling, tryptophan metabolism, tyrosine metabolism, ECM–receptor interaction, PI3K-Akt, Rap1, Ras, phagosome and pathogens infection-related, were shared in at least two groups out of NG, CG and SG (Figure 4 and Table S5). Among these, cytoskeleton-related proteins can help to maintain cell shape [24]; the focal adhesion-, tight junction-, proteoglycans- and glycosaminoglycans-related pathways are of critical importance in intercellular communication and cellular homeostasis in organisms, and play significant roles in forming the complex biomolecular structures that are necessary for insect survival, growth and development [19,20]; calcium signaling can mediate the environmental sensitivity of the diapause timer, and could be a key integrator of environmental condition (e.g., cold temperature) with downstream hormonal control of diapause [25]; both the tryptophan and tyrosine metabolism may contribute to color pattern in butterflies, and participate in resisting insecticides and defending against a wide range of pathogens, respectively [27,28]; other pathways, such as ECM–receptor interaction, PI3K-Akt, Rap1, Ras and phagosome, can functionally interact with each other to collectively make up an immune defense network [21,29]. These enhanced pathways imply strengthened cellular interactions and somatic maintenance, and improved structural defense and cellular immune response, which may strengthen the constitutive and inducible defenses against pathogen infection, as well as increase the stress resistance [30,31]. In contrast, ribosome, spliceosome, longevity and genetic information processing (e.g., DNA replication and repair, transcription, translation, etc.) pathways were commonly inhibited in at least two groups out of NG, CG and SG (Figure 4 and Table S5), suggesting the repression of cell replication and differentiation as the mechanism underlying the adults’ decelerated or arrested development status.

To decipher the shared signaling pathways among different insect groups, we further compared our enriched pathways with previously published genome-wide transcription studies of diapausing *D. melanogaster* [8]. The results show that almost 48% (23 out of 48) of the enriched pathways in that genome-wide transcription study were shared in our analysis. More importantly, the vast majority of these shared pathways in *D. melanogaster* were commonly enhanced (e.g., cytochrome P450, ECM–receptor interactions and metabolic-related pathways) or inhibited (e.g., genetic information processing, protein processing and circadian rhythm pathways), as shown in this study. We also compared our data to those of the cabbage butterfly *Pieris melete*, which is involved in summer and winter diapauses [32]. The results show that a series of signaling pathways related to diapause, such as calcium signaling, insulin signaling, forkhead transcription factor (FOXO), target of rapamycin (mTOR), mitogen-activated protein kinase (MAPK) and hormone-related signaling pathways, were shared between these two butterfly species (Table S5) (reviewed in [32]). The same or similar cases were also identified in other diapausing insect species, including the *Megachile rotundata* [33], *Locusta migratoria* [34], *Delia antiqua* [24], *Hyphantria cunea* [35] and *Drosophila suzukii* [36]. Overall, the overlap in enriched functional pathways was commonly enhanced or inhibited among *P. glacialis* populations and other diapausing insect groups, suggesting that the diapause-linked transcriptional regulation strategy of *P. glacialis,* accompanied by the success of colonization eastwards, enhance resistance to hostile conditions.

Interestingly, nutrient-sensing-related pathways, such as insulin (IS), mTOR and FOXO signaling pathways [37,38,39], were commonly inhibited, especially in NG and CG samples. The insulin signaling pathway can directly or indirectly interact with mTOR and FOXO signaling pathways to form an integrated nutrient-sensing network involved in the regulation of carbohydrate metabolism and energy restore [40,41]. The silencing of nutrient-sensing pathways may be causally related to reduced food intake, as well as to the arrested growth/development, enhanced stress response, increased lifespan, and other phenotypic changes characteristic of insect diapause [8,11,39,42]. In addition, cell cycle-, necroptosis- and apoptosis-related pathways were also systematically suppressed, implying a delayed life cycle, which results are similar to those for *Heterorhabditis* nematodes and *Drosophilia* flies [21,43].

**Figure 5:**
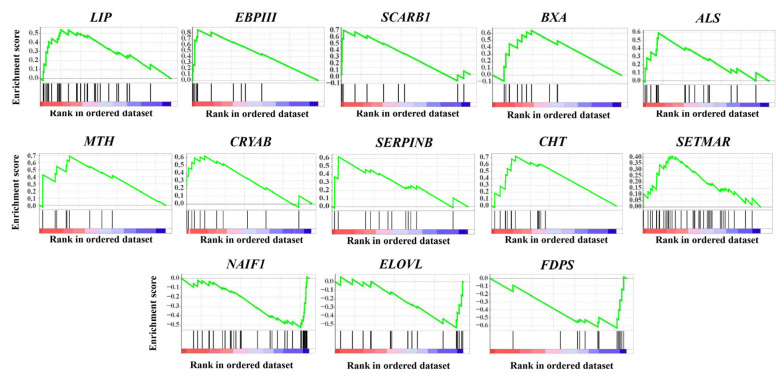
Enrichment plot of ten up-regulated and three down-regulated gene clusters based on gene set enrichment analysis. The upper portion of the plot shows the running enrichment score for the overall gene set. The lower portion of the plot shows where the members of the gene set appear in the ranked list of genes. More detailed gene information is available in the text.

Moreover, thirteen gene clusters were found to be enriched in multiple GSEAs based on pairwise comparisons to WG samples (Figure 5 and Table 1). Most of the up-regulated genes in each gene cluster were found to be enriched in NG and CG samples, which are directly or indirectly related to longevity regulation, immune defense and stress responses, whereas those down-regulated were mostly enriched in SG samples, mainly involved in fatty acids and hormone biosynthesis (Figure 5 and Table 1). Among these up-regulated genes, previous studies showed that *LIP* (lipase) can play a crucial role in fat catabolism responsible for oocyte maturation, sex pheromone biosynthesis and antiviral infection [44,45]; *EBPIII* (ejaculatory bulb-specific protein 3-like) in the chemosensory protein (CSP) gene family can function as receptors of environmental stimuli and in resistance to insecticides [46,47]; *SCARB1* (scavenger receptor class B member 1), an important regulator for cholesterol efflux and steroid hormone production, can also mediate phagocytosis and the antimicrobial peptide pathway in the endoparasitic wasps, involved in central nervous system (CNS)-mediated immune response [48,49]. Both *BXA* (bombyxin) and *ALS* (insulin-like growth factor-binding protein complex acid labile subunit) are involved in the insulin signaling pathway, and play important roles in the precise regulation of metabolism, growth, longevity and stress responses through functional interaction with each other [50,51]. Other genes, including *MTH* (G protein-coupled receptor Mth), *CRYAB* (crystalline alpha B), *SERPINB* (serpin B) and *CHT* (chitinase), have also been found to be mainly associated with longevity regulation, immune defense and stress responses in insects [30,52,53,54]. In addition, the enriched gene clusters with genes mostly down-regulated, including *ELOVL* (elongation of very long chain fatty acids protein) and *FDPS* (farnesyl diphosphate synthase), may participate in the unsaturated fatty acids biosynthesis of lipid metabolism, and in the formation of the juvenile hormone (JH) III in insect groups, respectively [55,56].

**Figure 6:**
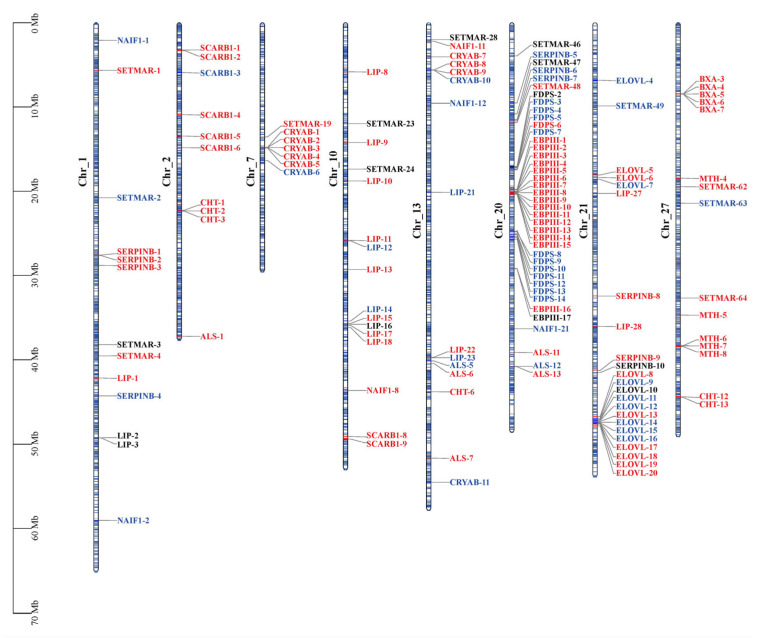
Eight representative chromosomal maps of *P. glacialis* with the distribution of the enriched gene clusters on the chromosome. Red, blue and black show up-regulated, down-regulated and non-changed (or not expressed) genes based on gene set enrichment analysis, respectively, with most of them harboring moderate expression changes. The left scale indicates the size of each chromosome. A complete chromosomal map of *P. glacialis* with the distribution of each enriched gene cluster is available in Figure S3.

Furthermore, duplicated genes (e.g., tandem duplications) were commonly found in the enriched gene clusters above, regardless of whether they were up- or down-regulated (Figure 6). Previous studies indicated that duplicated genes can be fixed by positive selection [57], and lead to novel expression patterns, as a mechanism of the genomic adaptation to a changing environment [58]. Notably, two transposon-derived gene clusters (transposable elements, TEs), *SETMAR* (Histone-lysine N-methyltransferase SETMAR) and *NAIF1* (nuclear apoptosis-inducing factor 1), were identified to be significantly enriched, with the former being mostly up-regulated in CG samples, while the latter were down-regulated in SG samples. *SETMAR*, a fusion gene previously found only in anthropoid primates comprising an N-terminal SET domain and C-terminal Hsmar1-derived (MAR) transposase [59], has been shown to function in DNA repair and enhance resistance to ionizing radiation, and would have contributed to the regulation of a vast gene expression network and epigenetic modification [60,61]. *NAIF1*, a domesticated transposase that originated from the ancestral Harbinger transposon, can induce apoptosis when overexpressed [60,62]. TEs are likely to be associated with gene expression variation and adaptive signatures in *Drosophila* [63], and also seem to be involved in the regulation of diapause in different insect groups [64,65].

### 2.4. Featured Modules Based on WGCNA

To obtain further insight into the habitat-specific adaptation mechanisms of *P. glacialis* populations resulting from diverged colonizing events [15], WGCNA was performed to investigate the co-expressional networks of all expressed genes. Two different subsets (WCN and WCS), reflecting transcription along two different dispersal routes, with each consisting of the normalized TPM values of WG and CG samples combined with those of either NG or SG samples, were used for analyses, respectively.

**Figure 7:**
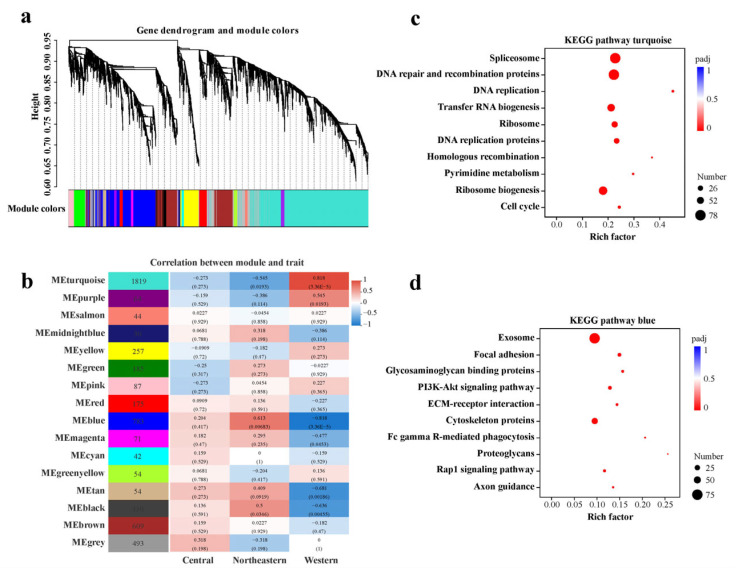
WGCNA of all expressed genes in the WCN dataset. (**a**) Hierarchical clustering tree (gene dendrogram) showing 16 modules of genes co-expressed by WGCNA. The major tree branches constitute 16 modules, labeled with different colors. (**b**) Module−locality relationship. Each row represents a module. Each column represents a specific sampling locality. The correlation coefficient between module and locality is represented by the value in each cell at the row−column intersection, with the *p*-value shown in parentheses. (**c**,**d**) KEGG enrichment analyses of the genes in the turquoise and blue modules, respectively. For each module, only the top ten pathways with the most significant enrichment are shown.

The analysis of the WCN dataset showed that these genes were clustered into 16 major modules (labeled with different colors; the gray module contains the remaining uncorrelated genes) (Figure 7a,b). Six modules (turquoise, blue, purple, magenta, tan and black) were significantly correlated with sampling localities, with high correlation coefficients (Figure 7b). Among them, the turquoise module contained 1819 genes and was highly positively correlated with the western area (correlation coefficient = 0.82, *p*-value = 3.4 × 10^−5^), while it was strongly negatively correlated with the northeastern area (correlation coefficient = −0.55, *p*-value = 0.02). In contrast, the blue module with 783 genes was highly positively correlated with the northeastern area (correlation coefficient = 0.61, *p*-value = 0.0068), but highly negatively correlated with the western area (correlation coefficient = −0.82, *p*-value = 3.4 × 10^−5^). Considering that these two modules contain the top two highest numbers of genes, both of which were also moderately correlated with the central area (|correlation coefficient| < 0.3, *p*-value > 0.05), they have been selected for further enrichment analysis.

For the WCN dataset, the KEGG enrichment analysis shows that genes in the turquoise module were primarily related to genetic information processing, protein processing, cell cycle and apoptosis regulation (Figure 7c and Table S7), which are strongly correlated with the cell replication, differentiation and aging processes underlying the developmental status. Most of the genes in this module were down-regulated in samples from the northeastern area (negative correlation) compared to those of the western area (Figure 7b), supporting the developmental arrest status of NG adult samples due to the general silencing of cell division and protein synthesis. In the blue module, the genes were significantly enriched for cell signaling and community, cytoskeleton regulation and immune defense against infections (Figure 7d and Table S7), suggesting that genes in this module were mainly responsible for somatic maintenance and defense system regulation to combat the pathogen infection and to increase the stress resistance. Most of the genes in this module were up-regulated in samples of the northeastern area (positive correlation) compared to those of the western area (Figure 7b), indicating the increased stress resistance and improved immune defense potential of NG samples. Meanwhile, 10 out of 16 modules were found to be moderately correlated with the central area, and the correlation coefficients between modules and sampling localities from the western to northeastern areas of China mostly changed with gradients (Figure 7b). This co-expression pattern probably implies that the transcriptional changes were more quantitative than qualitative between CG and NG samples, consistent with the overall statistics regarding DEGs, KEGG enrichment analyses and multiple GSEAs.

**Figure 8:**
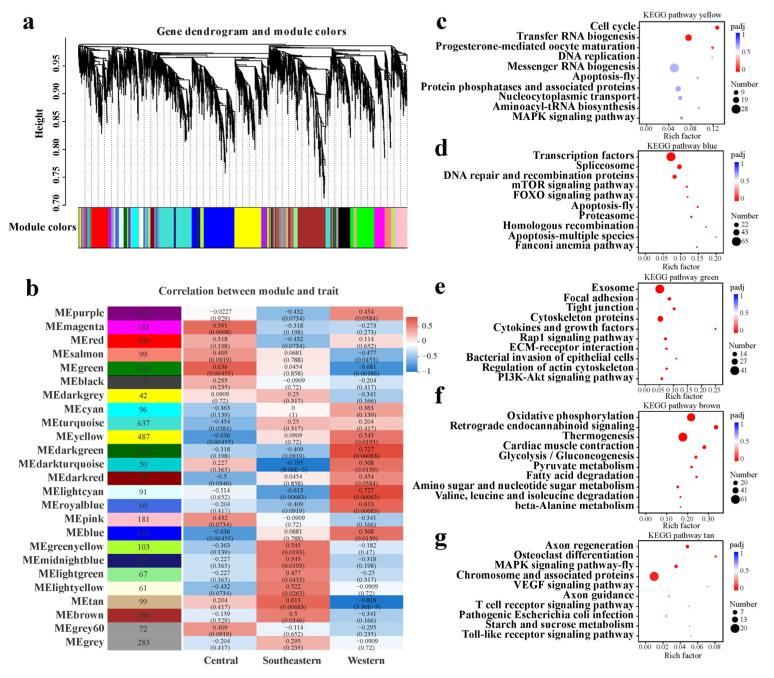
WGCNA of all expressed genes in the WCS dataset. (**a**) Hierarchical clustering tree (gene dendrogram) showing 25 modules of genes co-expressed by WGCNA. The major tree branches constitute 25 modules, labeled with different colors. (**b**) Module−locality relationship. Each row represents a module. Each column represents a specific sampling locality. The correlation coefficient between module and locality is represented by the value in each cell at the row−column intersection, with the *p*-value shown in parentheses. (**c**–**g**) KEGG enrichment analyses of the genes in the yellow, blue, green, brown and tan modules, respectively. For each module, only the top ten pathways with the most significant enrichment are shown.

In contrast, the analysis of the WCS dataset shows that these genes were clustered into 25 modules with relatively complex correlations between modules and sampling localities (Figure 8a,b), suggesting that the transcriptional changes are both quantitative and qualitative between CG and SG samples. Specifically, a total of 16 modules were significantly correlated with sampling localities (*p*-value < 0.05, Figure 8b). Among these, six modules (yellow, dark green, dark turquoise, light cyan, royal blue and blue) were positively correlated with the western area. Of these six modules, both the yellow and blue modules were also strongly negatively correlated with the central area, with the vast majority of genes primarily involved in the regulation of growth and development (e.g., pathways in genetic information processing, mTOR and FOXO), cell cycle and apoptosis (Figure 8c,d and Table S8). When compared to samples from the western area, most of the genes in these two modules were down-regulated in samples of the central area, suggesting adult developmental arrest in CG samples. Moreover, out of the two modules (magenta and green) most highly positively correlated with the central area, the green module also strongly negatively correlated with the western area, with most of the genes in this module highly expressed in CG samples (Figure 8e and Table S8). These genes are primarily related to cell signaling, cytoskeleton regulation and immune defense against infections, providing enhanced stress resistance and immune defense. Interestingly, among the other six modules (green-yellow, midnight blue, light green, light yellow, brown and tan) highly positively correlated with the southeastern area, the brown and tan modules contained 580 and 99 genes, respectively, and most of these genes were highly expressed in SG samples and enriched in the pathways of energy metabolism (e.g., oxidative phosphorylation, thermogenesis and fatty acid degradation), carbohydrate metabolism (e.g., glycolysis/gluconeogenesis, pyruvate metabolism and citrate cycle, etc.) and immune response (e.g., MAPK and cGMP-PKG signaling pathways) (Figure 8f,g and Table S8), thus contributing to rapid growth/development, as shown in the GSEA and the resulting earlier emergence time of SG samples compared to other samples.

### 2.5. RNA-Seq Validation Using RT-qPCR

**Figure 9:**
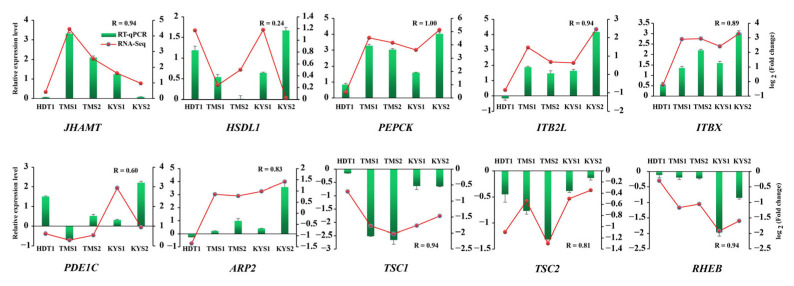
Validation expression patterns in *P. glacialis* representative samples determined by qPCR. Genes of the sample XLS1 were used for normalizing the relative expressions of the corresponding genes of the other five samples. Relative expression level was calculated using the 2^−ΔΔCt^ method. The left ordinate represents the qPCR-based expression levels and the right ordinate represents the RNA-seq-based expression levels. The error bar represents three repetitions.

In order to validate the RNA-seq, a total of ten genes (juvenile hormone acid O-methyltransferase, *JHAMT*; hydroxysteroid dehydrogenase-like protein 1, *HSDL1*; phosphoenolpyruvate carboxykinase, *PEPCK*; hamartin, *TSC1*; tuberin, *TSC2*; GTP-binding protein Rheb, *RHEB*; calcium/calmodulin-dependent phosphodiesterase 1C, *PDE1C*; actin-related protein 2, *ARP2*; integrin beta, *ITBX* and *ITB2L*) reported to be involved in diapause regulation in previous studies [8,11,24,35] were selected for testing using RT-qPCR. The results for six representative samples in four localities (XLS1, HDT1, KYS1, KYS2, TMS1 and TMS2) covering the most well-known marginal range of distribution in western, northeastern and southeastern China confirm the consistency of the gene expression pattern with overall high correlation (R = 0.88, *p*-value = 2.7 × 10^−17^) (Figure S4). The correlations between RNA-seq and qPCR data were extremely strong for eight genes (R > 0.80), while the correlations were less strong for the other two genes (with a range of R values from 0.20 to 0.60) (Figure 9). We speculate that these discrepancies could be related to the methodological difference between RNA-seq and qPCR, which seemed to be common in the transcript-level analyses. Nonetheless, the variation tendencies in the RNA-seq data curve and the qPCR histogram are mostly similar, suggesting our RNA-seq and qPCR data analyses are reliable.

## 3. Discussion

As global warming is increasingly exacerbating, rapid climate changes may lead to shifts in species’ ranges, population declines, and even extinctions [66]. In response, diapause can occur at different ontogenetic stages (e.g., in egg, embryonic, larval, pupal or adult stage) in different insect species under various environmental contexts, but usually in a single specific stage for each species [11,32,35,43,64]. In addition, diapause could evolve very rapidly and polyphyletically in different insect lineages (reviewed in [11]). Hence, previous studies have identified different transcriptional patterns of diapause at the gene level among different insect lineages. However, the genetic toolkit of diapause is likely to be observable in the activation/inhibition of the common functional pathways regulating the hallmark diapause-linked phenotypes [11,32]. Therefore, in the present study, we focused mainly on the enriched functional pathways/gene sets to compare the transcriptional patterns observed in *P. glacialis* populations with analogous patterns published in other insects. Several strategies for large-scale transcriptomic interpretation, such as DEG enrichment analysis, GSEA and WGCNA, were used to investigate the featured pathways/gene set to minimize the disadvantages of individual methods, and to characterize the genome-wide expression patterns for different *P. glacialis* populations. Substantial functional pathways underpinning the diapause-linked phenotype characteristics, which could contribute to the success of *P. glacialis’* colonization out of the QTP from western to eastern China, were herein identified for the first time [15].

A few key functional pathways, including the evolutionarily conserved hormone (endocrine system), insulin-IGF (IIS) and mTOR-related signaling pathways, have been shown to be implicated as key regulators of insect diapause that promote local adaptation [8,11,67]. Among these, hormone-related pathways such as ecdysone (a steroid hormone) and juvenile hormone (JH) have been implicated in reproduction, stress responses and longevity regulation, and play key roles in insect diapause [8,35,67,68]. In the present study, a series of enriched pathways (e.g., cholesterol metabolism and cytochrome p450, etc.) and one gene cluster (*SCARB1*) involved in steroid hormone production were found to be uniquely enhanced in NG samples. Among them, both cholesterol metabolism and cytochrome p450 can participate in 20-hydroxyecdysone (20-HE, the active form of ecdysone) biosynthesis [43]. In contrast, both the enriched pathway related to steroid hormone biosynthesis and the gene cluster *FDPS* responsible for the formation of the JH III were shown to be significantly inhibited in SG samples (Figure 2d and Figure 5). This result suggests that the hormonally regulated state of diapause was likely to be different among the adult *P. glacialis* populations. Given the fact that the specific hormones and their levels are individual-, species- and diapause stage-dependent [8,24,43,69,70], the hormone-inducing diapause regulation patterns among *P. glacialis* populations deserve detailed attention in future functional studies.

The silencing of the IIS and mTOR signaling pathways is related to the suppression of growth/development, enhanced stress response and extend lifespan in several diapausing insect groups and nematode *Caenorhabditis elegans* [8,11,39]. In the current study, functional pathways linked to IIS and mTOR were generally suppressed, especially in NG and CG samples. The gene expression levels of several core molecular components in these two pathways (e.g., *PEPCK*, *TSC1*, *TSC2* and *RHEB*) were validated via qPCR (Figure 9). The increased expression of *PEPCK*, a potential marker for distinguishing between diapause and direct development [71], can enhance gluconeogenesis, as was also found in SG samples herein and other diapausing insects [8]. It is worth noting that several other IIS-related pathways (e.g., insulin secretion, FOXO and relaxin signaling pathway) and two gene clusters (e.g., duplicated genes *BXA* and *ALS*) were concurrently enhanced or inhibited (Figure 5 and Table S5), and this probably indicates the concomitant up-regulation of some positive and negative components of IIS-related pathways for precise regulation during the phase of diapause. A similar regulation pattern was also described in the diapausing flies [8,11].

Under the hormonal control and regulation of the key signaling pathways above, a series of other pathways related to diapause regulation have been well presented in our enrichment analyses, such as those involved in genetic information processing, cell signaling, metabolism, immunity, stress response, cell cycle and aging, etc. These enriched pathways could interact with each other and form a series of complex regulatory networks (reviewed in [8]), supplying new evidence that complex, polygenically expressional variation may be involved in adaptive regulation in diapause, which is likely responsible for the successful colonization of new habitats by *P. glacialis* populations.

In the present study, both the DEG enrichment analysis and GSEA results indicate that enriched pathways related to cell signaling, the immune system and the endocrine system were mostly enhanced, while those related to genetic information processing were generally inhibited in NG samples regardless of whether they were compared to WG or CG samples (Figure 2, Figure 3 and Figure 4 and Table S5). In contrast, enhanced genetic information processing- but inhibited cell signaling-related pathways were found in SG samples relative to CG samples, different from the enrichment results of other pairwise comparisons. Furthermore, a series of duplicated genes (gene clusters including TEs) functioning in germ cell maturation, hormone or sex pheromone biosynthesis, immune defense and longevity regulation, important to survivability, growth and reproductive capacity, were identified to be mostly up-regulated in NG and/or CG samples. It is worth mentioning that one of the TEs herein, *SETMAR*, has been shown to contribute to the emergence of new gene regulatory networks, in that a modest overexpression of *SETMAR* can lead to the misregulation of 1500 genes [61]. However, only three gene clusters functionally different from those enriched in CG and NG samples were identified to be significantly enriched in SG samples in the present study (Table 1). Though moderate expression changes were found for the vast majority of duplicated genes herein, the co-expression pattern showing most genes commonly up- or down-regulated probably indicates a transcriptional pattern for *P. glacialis* populations in diapause under environmental stress conditions, which maybe provide some insights into how adaptation to environmental changes influences duplicated gene expression [58,72], although further investigations are needed.

Moreover, the results of our WGCNA also reveal that the featured modules were significantly correlated with sampling localities underlying the habitat-specific adaptability of different *P. glacialis* populations. The signaling pathways enriched in the featured modules were found those responsible for immune defense, stress resistance and somatic maintenance in the CG samples (Figure 8), which could contribute to the success of colonization from western to central China. This transcriptional regulation pattern seemed to be strengthened with the successive colonization from central to northeastern China. Though a substantial fraction of the enriched signaling pathways underpinning the diapause-linked phenotype were shared among SG, CG and NG samples, many enhanced metabolism- and immune defense-related pathways were uniquely enriched in SG samples compared to CG samples (Tables S5 and S8), implying the importance of metabolic and immune regulation for successive colonization from central to southeastern areas.

Based on the results above, it is reasonable to speculate that *P. glacialis* populations, especially in central to northeastern China, have probably evolved several remarkable adaptive characteristics accompanied by their dispersal out of the QTP, including higher hormone biosynthesis levels, stronger somatic maintenance and more sensitive responses to environmental stimuli than populations in southeastern China. On the other hand, for *P. glacialis* populations in southeastern China, the decreased ecdysone and/or JH level can induce or promote reproductive arrest, slowing aging and long-range migration [67,68]. Thus, we speculate that adult *P. glacialis* populations in southeastern China are more similar to migrant adults of monarch butterfly [68], and could temporarily suspend reproduction in response to environmental stress (e.g., relatively higher annual mean temperature and precipitation in southeastern than in northeastern China, as shown in Figure S5). Moreover, under the regulation of upstream signaling pathways (e.g., 20HE, JH and IIS) [67], they probably utilize the lipids and glycogen energy reserves stored in their body for their survival and growth. The results of GSEA and WGCNA confirm that various unique pathways mainly responsible for energy metabolism were significantly enhanced in SG samples, including oxidative phosphorylation, thermogenesis, fatty acid degradation, glycolysis/gluconeogenesis, amino sugar and nucleotide sugar metabolism, and glyoxylate and dicarboxylate metabolism (Figure 8f and Table S5). All these pathways can catabolize energy reserves, such as fatty acids, glucose and other sugar, to generate ATPs for flight and survival [64,73,74]. The glyoxalate pathway has been known to be important in dauer stages of *Caenorhabditis elegans*, and has also been reported in the infective juvenile stage of entomopathogenic nematodes [21]. In addition, a number of synergistically enhanced pathways with functions related to microbial defense and immune response (e.g., retrograde endocannabinoid signaling, toll-like receptor and NF-κB signaling pathway) were also identified in GSEAs, all of which may have contributed to the adaptation of *P. glacialis* in southeastern China. Together, combined with the overall statistics of DEGs, KEGG enrichment analysis, GSEA and WGCNA, all our results consistently reveal the habitat-specific adaptability of different *P. glacialis* populations, and also suggest that the genome-wide expression patterns of the CG samples were more similar to those of NG samples than to SG samples, which is consistent with the results of population genetics analyses based on the genotyping-by-sequencing (GBS) data of our recent study [15].

## 4. Materials and Methods

### 4.1. Sample Collection

*P. glacialis* imago individuals (*n* = 24) were collected from eight localities, covering nearly the entire known range of distribution in China. For each locality, all sampling was performed at the same time during the day, between 10:00 and 13:00, to avoid the potential effect of circadian variability on the expression profiles. According to the meteorological data of the sampling locality (Figure S5) and the geographical dispersal pattern of *P. glacialis* as shown previously [15], the samples here were divided into four groups, classified as the western group (WG), central group (CG), northeastern group (NG) and southeastern group (SG), respectively (Figure 1 and Table S1). All samples were initially preserved in RNA stabilization solution (Sangon Biotech, Shanghai, China) in the field and transferred to −80 °C until RNA extraction. Muscle tissues from the thorax of three individuals per sampling locality were used for purified RNA extraction.

### 4.2. mRNA-Seq Library Construction and Illumina Sequencing

Library construction and Illumina sequencing were performed following the methods in the previous study [13]. The sequencing library was paired-end-sequenced using the Illumina HiSeq 2500 platform (Shanghai Personal Biotechnology Co. Ltd, Shanghai, China). After the adaptor contamination was removed, the reads were screened to trim the bases with a quality score of Q < 20 using 5-bp windows, and reads of less than 50 bp and ambiguous nucleotides were removed.

### 4.3. Mapping, Transcript- and Gene-Level Abundance Estimation

After quality filtering, all the remaining reads were collected and then mapped to the *P. glacialis* genome sequence to acquire the genes for each sample using HISAT2 v2.1.0 [75] and StringTie v2.1.5 [76]. The assembled transcripts of each sample were merged using StringTie v2.1.5 –merge, creating an updated annotation file for the *P. glacialis* genome. Transcript abundances were estimated using StringTie v2.1.5 with the parameters –e and –B. A gene-level read count matrix was generated using the prepDE.py script provided as part of the StringTie package.

### 4.4. Expression Level Normalization and Differential Expression Analysis

A pipeline was set up to normalize the data for all samples. Firstly, TPM (transcripts per million) values for each gene were calculated based on the read count and exon length using TBtools [77], the sum of which for all genes in each sample was one million. Secondly, the online tool of Majorbio Cloud Platform was used to remove batch effects [78], and then the expression values were normalized by the scaling procedure as previously described [79,80]. Specifically, among the genes with expression values in the interquartile range (25–75%) in terms of average expression levels across samples, a total of 1000 genes with the most conserved ranks across all samples were identified, and their median expression levels were assessed in each sample. Finally, scaling factors that adjust these medians to a common value were derived, and were then used to scale the expression values of all genes in the samples [79]. The resulting gene expression values were used for downstream analyses. Moreover, TPM values without normalization, the sum of which was one million for each sample, were also used for subsequent analyses for comparison.

Differential gene expression analysis between groups was performed to reveal the long-term habitat-specific adaptation mechanisms. Thus, five differentially expressed gene datasets, derived from comparisons of the NG vs. WG, SG vs. WG, CG vs. WG, NG vs. CG, and SG vs. CG groups, were focused on. Genes with a change in expression level satisfying |log2FoldChange| ≥ 1 and Benjamini–Hochberg adjusted *p* value < 0.05 were defined as DEGs using DESeq2 and edgeR based on the read counts, with DESeq2 using the relative log expression (RLE) normalization and edgeR using the trimmed mean of M values (TMM) normalization [81,82]. The shared DEGs obtained from DESeq2 and edgeR in each pairwise comparison were retained for further analyses to reduce the false positives. Subsequently, KEGG enrichment analyses were performed to determine the biological features of these DEGs with TBtools, using the adjusted *p* value < 0.05 as the cutoff value [77]. In addition, principal component analysis (PCA) was conducted to reveal the clustering effects in the transcriptomic profiles of all groups.

### 4.5. Gene Set Enrichment Analysis (GSEA)

Multiple gene set enrichment analyses (GSEAs) for pairwise comparisons were performed based on the normalized and non-normalized TPM values, respectively, using the GSEA v4.2.3 software [83]. To decipher gene function enrichment at different levels, gene sets for GSEA were defined using KEGG orthology (KO) identifiers and pathway categories, respectively, and the defined genes from the whole genome were here used as the default background distribution. Gene sets with a normalized enrichment score (|NES|) >1, *p*-value < 0.05 and FDR < 0.25 were considered statistically significant [83]. The commonly enriched KEGG pathways among different comparisons were visualized using the module EnrichmentMap using Cytoscape software [84,85].

For each significantly enriched gene set defined using the KO identifier, the following procedure was also used to verify the members in each gene cluster in the *P. glacialis* genome. The published representative homologous proteins in Insecta (including *Drosophila melanogaster*, *Bombyx mori*, *Apis mellifera*, *Danaus plexippus*, *Papilio machaon*, *Papilio bianor*, *Pieris rapae*, etc.) downloaded from the National Center for Biotechnology Information (NCBI, https://www.ncbi.nlm.nih.gov (accessed on 12 October 2022)) or InsectBase (http://www.insect-genome.com (accessed on 12 October 2022)) were used as queries to search against the genome of *P. glacialis*, using the BLASTP algorithm (E-value < 10^−5^). The genes with identities lower than 30% were filtered out, and then subjected to InterPro (www.ebi.ac.uk/interpro (accessed on 15 October 2022)) to confirm the presence of the conserved domain [86]. After removing redundancies, the top hits for putative genes were retained.

### 4.6. Weighted Gene Co-Expression Network Analysis (WGCNA)

Weighted gene co-expression network analysis (WGCNA) can be used as a data exploratory tool to identify highly interconnected gene clusters in different modules across samples using unsupervised clustering [87]. In this study, WGCNA was used to identify key gene clusters and then correlate their expression patterns to the sampling locality, so as to reveal the habitat-specific adaptation mechanisms of *P. glacialis* underlying the extraordinary adaptability to local seasonal environmental challenges, with the following parameters: unsigned for TOMType, 30 for minModuleSize, 0.35 for mergeCutHeight, and default values for the other parameters.

### 4.7. Validation of Gene Expression by Real-Time RT-qPCR

To validate the expression patterns derived from our RNA-Seq analysis, ten genes, including both DEGs and non-DEGs among the representative samples covering the most well-known marginal range of distribution in western, northeastern and southeastern China, were selected for real-time reverse transcription (RT) quantitative PCR analysis (primers were listed in Table S2). Reversed cDNA was synthesized using the PrimeScript™ 1st stand cDNA Synthesis Kit (Takara, Shanghai, China) from total RNA isolated as described above. All RT-qPCR experiments were run in triplicate using the LightCycler 480 II (Roche Diagnostics, Basel, Switzerland) with SYBR green (Vazyme, Nanjing, China) with the following cycling parameters: 95 °C for 5 min, and 40 cycles of 95 °C for 15 s, 60 °C for 30 s. The amplification and detection of only one PCR product was confirmed using melting curve analysis of the amplification products at the end of each PCR. The expression levels of different genes were analyzed using the comparative CT method (2^−ΔΔCt^ method) [88]. To ensure the robustness of the reference gene, the gene expression stability of commonly used housekeeping genes under biotic conditions was evaluated according to previous studies [89], and the elongation factor 1 alpha (*EF1-α*) gene was finally chosen as the reference gene. The R values of the Spearman’s correlation coefficient were calculated to represent the correlations between the data obtained from the qPCR and RNA-seq.

### 4.8. Statistical Analysis

Correlation analysis, hierarchical clustering, and principal component analysis (PCA) were performed using the SPSS software (version 23.0; IBM Inc., Chicago, IL, USA) or the online tool of Majorbio Cloud Platform [78]. The nonparametric method with Kruskal–Wallis or Wilcoxon signed ranks test was also conducted using the SPSS software. In all analyses, statistical significance was shown by a *p*-value of less than 0.05.

## 5. Conclusions

In the present study, we profiled relatively large-scale transcriptomics of different geographic populations of *P. glacialis* in China via several strategies for data interpretation. Based on the stringent screening criteria of DEGs and gene function enrichment at different levels, with different groups (WG or CG samples) used as the control, all results consistently indicate that substantial pathways involved in immune response, metabolic processes, cell signaling, developmental processes, reproduction, transcription, translation, protein processing, cell cycle and aging are shared with those revealed in studies of diapausing insect groups, most of which are commonly enhanced or inhibited, thus underpinning the diapause-linked phenotype of the adult *P. glacialis* populations in central to eastern China. Moreover, different gene enrichment patterns were also revealed, probably suggesting the habitat-specific adaptability of different *P. glacialis* populations. In addition, a suite of duplicated genes (including two transposable elements) with co-expression patterns could promote the plastic responses of *P. glacialis* to different environmental challenges. Taken together, our data provide a population-wide and comprehensive analysis of transcriptional changes implying the diapause-like status of geographic populations of *P. glacialis* in central to eastern China, and show the utility of this mountain butterfly species as a model to analyze the genetics of diapause and its effects on adaptation to heterogeneous environmental conditions.

## Figures and Tables

**Table 1:** List of the enriched gene clusters identified in *P. glacialis* populations and related information.

Enriched Group	Gene Name	Description	Main Function
CG and NG	*LIP*	Lipase	Fat catabolism
CG and NG	*EBPIII*	Ejaculatory bulb-specific protein 3-like	Chemoreception and resistance to insecticides
CG and NG	*BXA*	Bombyxin	Metabolism, growth and longevity regulation
CG and NG	*CHT*	Chitinase	Digestion, molting and immune defense
NG	*SCARB1*	Scavenger receptor class B member 1	Steroid hormone production and immune defense
NG	*CRYAB*	Crystalline alpha B	Stress responses and extended lifespan
NG	*SERPINB*	Serpin B	Immune defense
NG	*ALS*	Insulin-like growth factor-binding protein complex acid labile subunit	Metabolism, growth and longevity regulation
CG	*MTH*	G protein-coupled receptor Mth	Chemoreception and extended lifespan
CG	*SETMAR*	Histone-lysine N-methyltransferase SETMAR	DNA repair and epigenetic modification
SG	*NAIF1*	Nuclear apoptosis-inducing factor 1	Apoptosis regulation
SG	*ELOVL*	Elongation of very long chain fatty acids protein	Long-chain fatty acids biosynthesis
SG	*FDPS*	Farnesyl diphosphate synthase	Juvenile hormone production

## Data Availability

Both the genome and transcriptome sequencing data were deposited into GenBank with the BioProject numbers PRJNA893814 and PRJNA916644, respectively. The datasets used and/or analyzed during the current study are available from the corresponding author upon reasonable request.

## Supplementary Materials

The following supporting information can be downloaded at: https://www.mdpi.com/article/10.3390/ijms24065577/s1.
