# Supplementary material for: Diapause-Linked Gene Expression Pattern and Related Candidate Duplicated Genes of the Mountain Butterfly Parnassius glacialis (Lepidoptera: Papilionidae) Revealed by Comprehensive Transcriptome Profiling
Source: Int J Mol Sci. 2023 Mar 14;24(6):5577. doi: 10.3390/ijms24065577 (PMC10058462; doi:10.3390/ijms24065577)
Supplement: Supplementary file 1 [file ijms-24-05577-s001.zip › Supplementary Files/Supplementary Figure S1-S5.pdf]

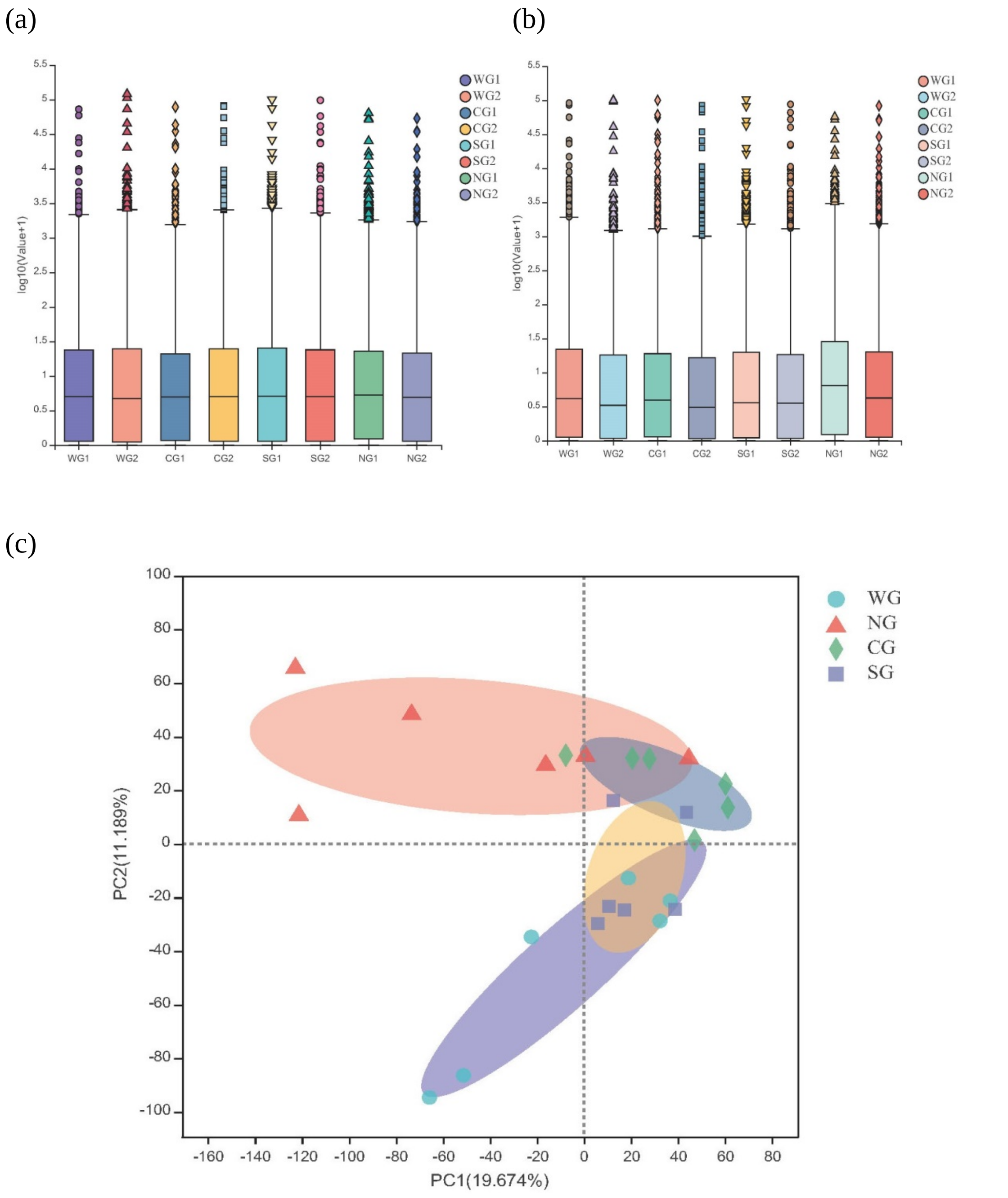

Figure S1. Boxplots and the scatter plot of principal component analysis (PCA) among groups. (a) Boxplot for normalized dataset. (b) Boxplot for non-normalized dataset. (c) The scatter plot of principal component analysis (PCA) among groups. WG, western group; CG, central group; NG, northeastern group; and SG, southeastern group (similarly hereinafter).

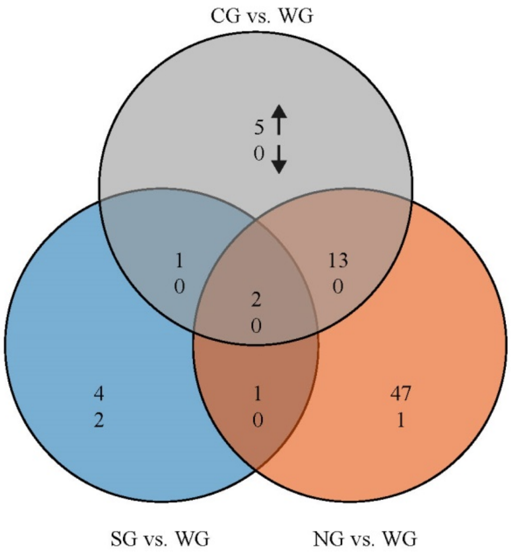

Figure S2. Venn diagrams showing overlaps of enriched KEGG pathways of the DEGs enhanced (upward arrow) or inhibited (downward arrow) in five pairs of comparisons.

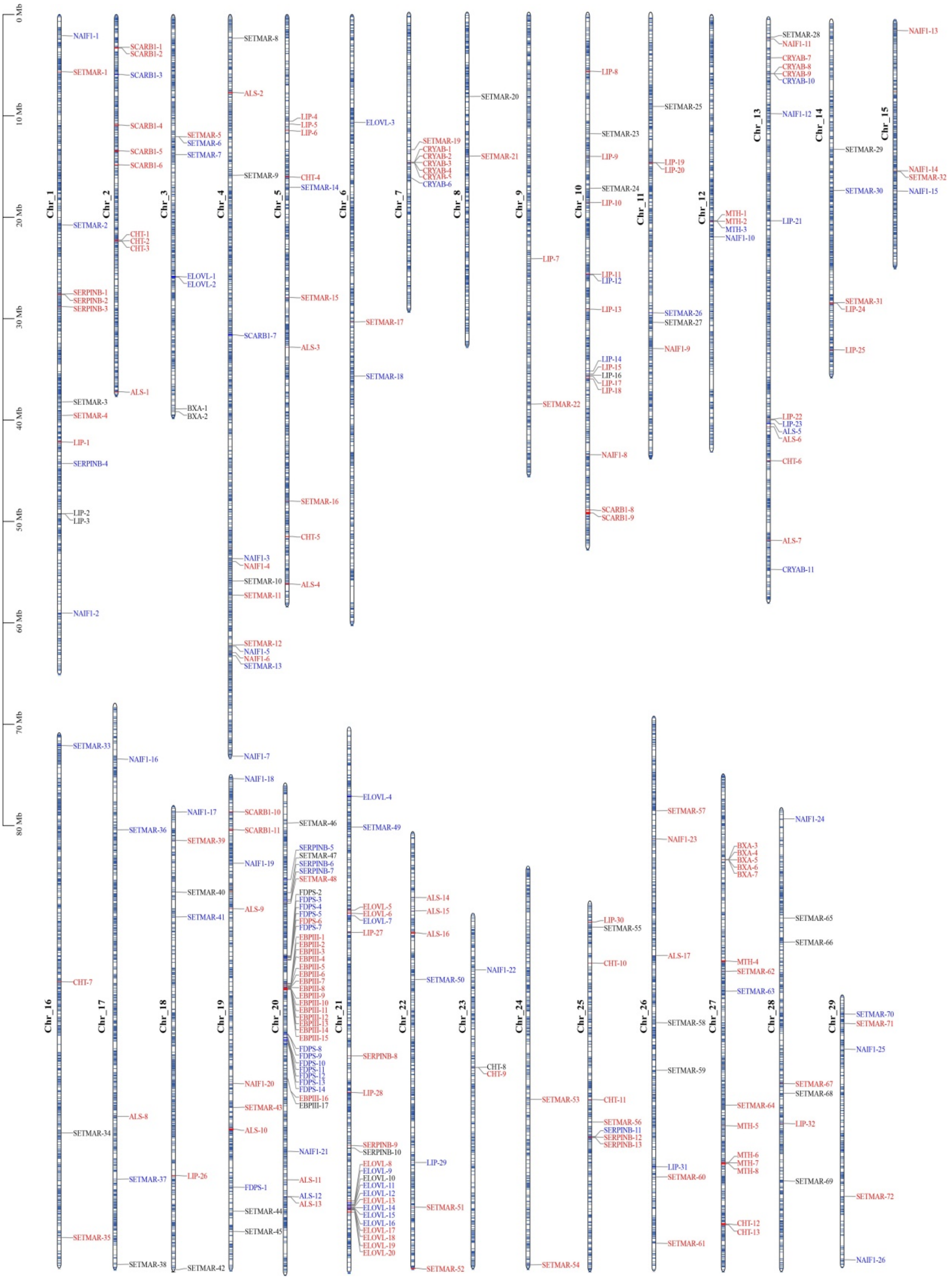

Figure S3. Chromosomal map of *P. glacialis* with the distribution of all enriched gene clusters. Genes in red, blue and black color show up-regulated, down-regulated and non-changed (or not expressed) ones based on gene set enrichment analysis, respectively, with most of them harboring moderate expression changes. The left scale indicates the size of each chromosome.

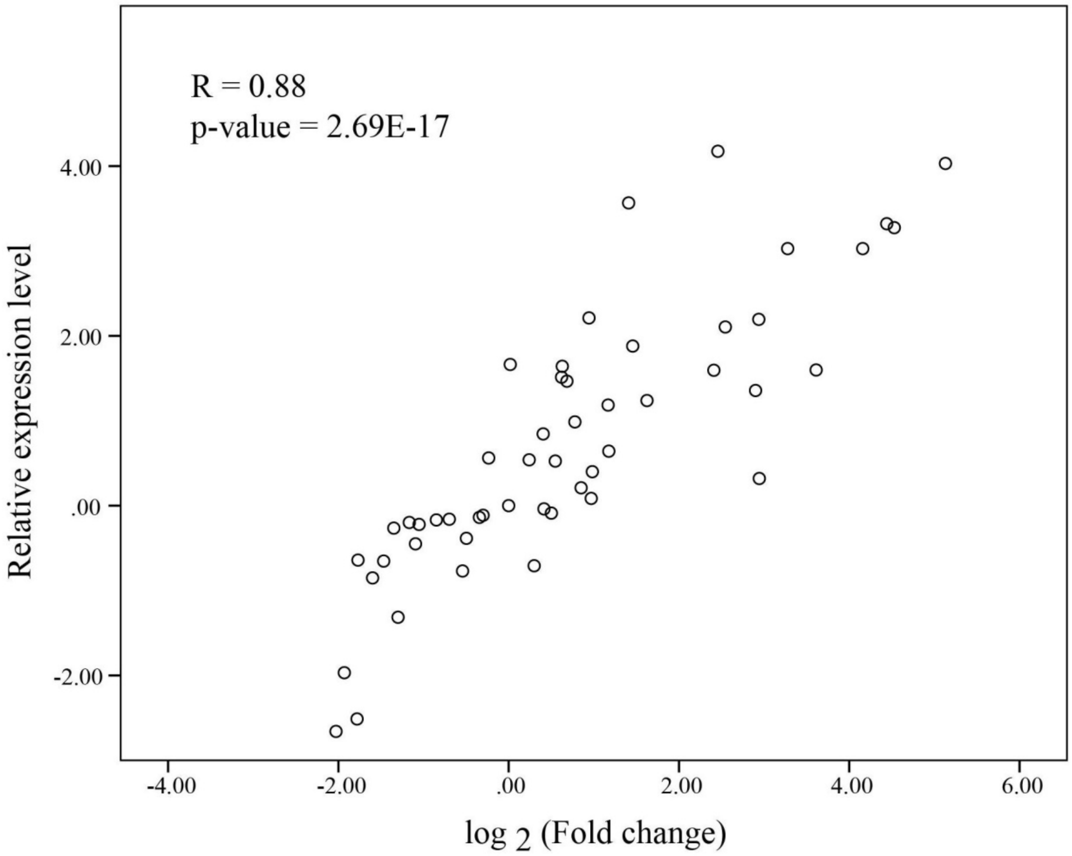

Figure S4. Validation expression pattern in *P. glacialis* representative samples by qPCR reflecting the overall high correlation between RNA-seq and qPCR data. Relative expression level was calculated using the $2^{-\Delta\Delta Ct}$ method. The y-axis represents the qPCR-based expression levels and the x-axis represents the RNA-seq-based expression levels.

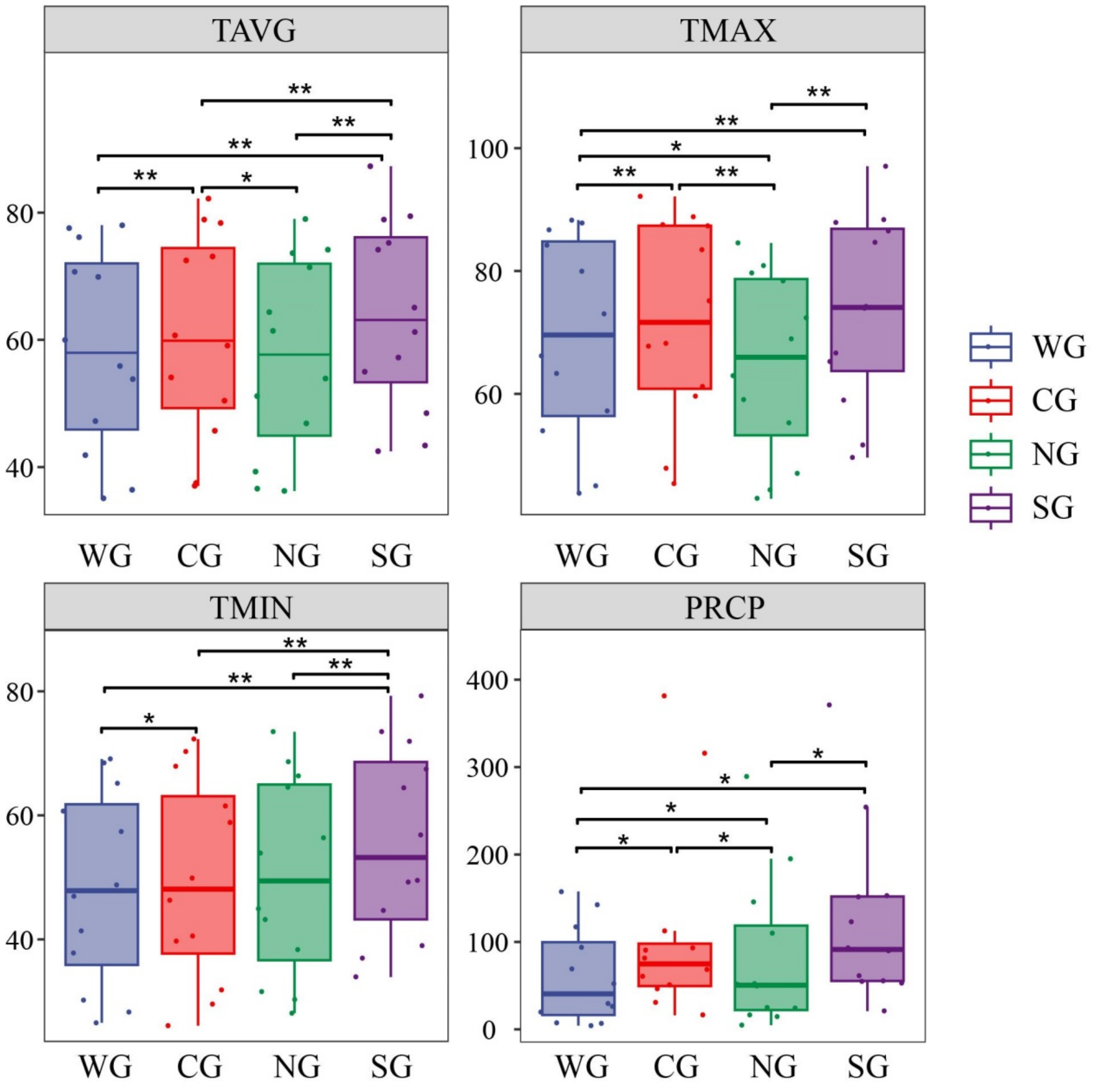

Figure S5. Statistics of the meteorological data of the sampling locality derived from the global summary of the month (GSOM) data files (accessed at the location https://www.ncei.noaa.gov/data/gsom/). TAVG, average monthly temperature given in the degree Fahrenheit, computed by adding the unrounded monthly maximum and minimum temperatures and dividing by 2; TMAX, monthly maximum temperature given in the degree Fahrenheit, being equal to the average of daily maximum temperature in each month; TMIN, monthly minimum temperature given in the degree Fahrenheit, being equal to the average of daily minimum temperature in each month; PRCP, total monthly precipitation given in millimeters. The level of significance was shown by the p-value of Wilcoxon signed ranks test (*, $P < 0.05$; **, $P < 0.01$).
